# Contribution of neuropsychiatric symptoms in Parkinson’s disease to different domains of caregiver burden

**DOI:** 10.1007/s00415-021-10443-7

**Published:** 2021-02-25

**Authors:** L. M. Chahine, R. Feldman, A. Althouse, B. Torsney, L. Alzyoud, S. Mantri, B. Edison, S. Albert, M. Daeschler, C. Kopil, C. Marras

**Affiliations:** 1 Department of Neurology, University of Pittsburgh School of Medicine, University of Pittsburgh, 3471 Fifth Avenue, Kaufmann Medical Building, Suite 811, Pittsburgh, PA 15213 USA; 2 Center for Clinical Trials and Data Coordination, Division of General Internal Medicine, University of Pittsburgh School of Medicine, Pittsburgh, PA USA; 3 Temple University College of Education and Human Development, Philadelphia, PA USA; 4 Department of Neurology, Duke University School of Medicine, Durham, NC USA; 5 Behavioral and Community Health Sciences, Graduate School of Public Health, University of Pittsburgh, Pittsburgh, PA USA; 6 Columbia University, Columbia School of Social Work, New York, NY USA; 7 The Michael J. Fox Foundation for Parkinson’s Research, New York, NY USA; 8 University of Toronto, University Health Center, Toronto, Canada

**Keywords:** Caregiver burden, Parkinson’s disease, Neuropsychiatric symptoms, Caregiving

## Abstract

**Introduction:**

Caregiver burden is high among caregivers of PD patients (CPD). Neuropsychiatric symptoms are leading contributors to CPD burden, but whether different symptoms differentially impact domains of caregiver burden is not known. Our objective was to examine which neuropsychiatric symptoms and demographic factors contribute to different domains of caregiver burden in PD.

**Methods:**

This was a cross-sectional online survey study. Participants were recruited from the Fox Insight (FI) study and were eligible if they identified themselves as a CPD. The primary outcome was the Caregiver Burden Inventory (CBI) total score and its 5 sub-domain scores. The Neuropsychiatric Inventory Questionnaire (NPI-Q) assessed caregiver-reported neuropsychiatric symptoms in the care recipient. Multivariable linear regression models were used to characterize the associations between NPI-Q symptom severity scores and CBI scores. Covariates were caregiver age, sex, education, and caregiving duration.

**Results:**

The sample consisted of 450 CPD, mean age 65.87 (SD 10.39) years, 74% females. After adjusting for covariates, CBI total score was predicted by NPI-Q total score (*β* = 1.96, *p* < 0.001); model adjusted *R*^2^ = 39.2%. Anxiety severity had the largest effect size [standardized *β* (s*β*) = 0.224] on the time-dependency domain, which was also associated with female sex (s*β* = − 0.133) and age (s*β* = 0.088). Severity of disinhibition (s*β* = 0.218), agitation (s*β* = 0.199), and female sex (s*β* = 0.104) were associated with greater emotional burden.

**Conclusion:**

Our findings indicate that demographic characteristics and specific neuropsychiatric symptoms contribute differentially to domains of caregiver burden. Tailored interventions to support CPD are needed.

**Supplementary Information:**

The online version contains supplementary material available at 10.1007/s00415-021-10443-7.

## Introduction

Parkinson’s disease (PD) is the second most common neurodegenerative disorder, and its prevalence is increasing worldwide [1, 2]. PD is marked by inexorable progression of motor and non-motor symptoms over the course of the disease. As the disease progresses, individuals require increasing assistance with activities of daily living [3]. Informal caregivers provide the majority of care and support for individuals with PD in the United States [4]. Caregiver burden—the negative consequences of caregiving on the caregiver—is high among caregivers of PD patients (CPD), and can adversely affect the physical and mental health of both the caregiver and the PD patient [5]. Therefore, understanding the contributors to caregiver burden in PD is critical.

PD manifestations in the care recipient (the PD patient whom the caregiver is providing care for) are a key determinant of caregiver burden in PD. While motor severity and motor complications in PD have some contribution to caregiver burden, non-motor symptoms have a greater impact. Indeed, multiple studies from diverse populations have shown that neuropsychiatric symptoms, including psychosis, apathy, depression, and dementia, are the leading contributors to caregiver burden in PD [6–11]. These neuropsychiatric symptoms in the care recipient could be assessed via physician, patient, and/or caregiver report. Given that caregiver *perception* of disease manifestations and severity in the care recipient could have a strong influence on caregiver burden, examining the relationship between caregiver-reported neuropsychiatric manifestations of PD in the care recipient and measures of caregiver burden is crucial [12–14].

Caregiver burden is multidimensional, encompassing physical, emotional, and financial aspects [15], among others. Most studies of caregiver burden in PD have used global measures, and the contributors to different domains of caregiver burden in PD are not known. Given the established strong contribution of neuropsychiatric symptoms to caregiver burden of CPD [6–9], understanding whether different neuropsychiatric symptoms also differentially impact domains of caregiver burden is important towards providing meaningful, personalized support to CPD. Towards the latter, determining whether caregiver characteristics also have differential impacts on each domain is important and, to our knowledge, has been minimally studied in PD. We aimed to examine, among a large cohort of CPD, which caregiver demographics and caregiver-reported neuropsychiatric symptoms in the care recipient contribute to different domains of caregiver burden in PD.

## Methods

### Sample

**Fig. 1:**
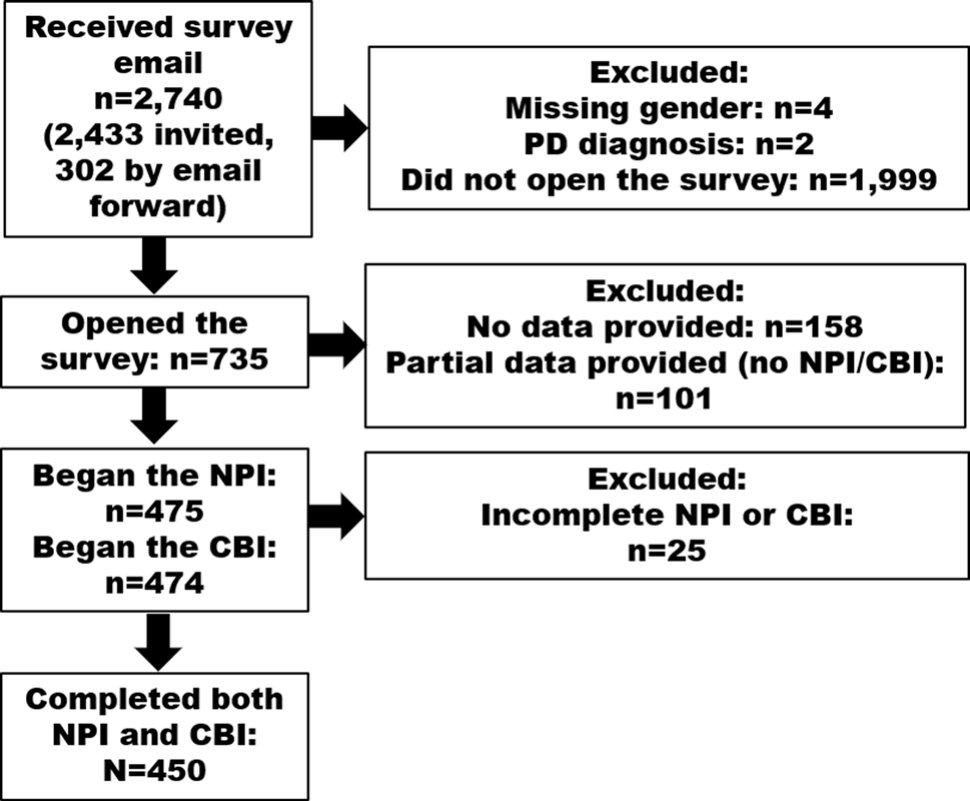
Flow diagram of potentially eligible and final studied sample

This was a cross-sectional study that was carried out as part of a research program investigating caregiver burden in PD [16, 17]. Participants were recruited from the Fox insight (FI) study, an online-only study in which individuals with and without self-reported PD participate in online assessments [18]. FI participants were considered eligible to participate in this study if they had identified themselves as being caregivers of patients with PD. An email invitation was sent to eligible individuals, and those clicking on a link in the email went on to receive the survey. Individuals could also participate if they: (1) were forwarded the email invitation, (2) enrolled in FI after the email invitation was sent, they could view the survey and opt to click on it for potential participation. Only individuals completing all items on the neuropsychiatric symptoms and caregiver burden questionnaires were included in this analysis (see below; Fig. 1).

### Assessments

- Demographics—caregiver age, sex, education
- PD disease duration in care recipient
- Caregiver role—a series of questions determined if the respondent was the primary caregiver, was employed outside the home, was paid, and if they lived with the patient.

To evaluate caregiver responsibilities, participants were asked to select all tasks that applied from the following: assisting with personal care (e.g., helping with bathing, grooming, dressing, etc.), food preparation, obtaining and/or administering prescribed medications, general health care (such as scheduling medical appointments, making sure they get to appointments, etc., but does not include medications), mobility assistance (e.g., helping them getting up from a chair, assisting with balance), providing emotional support, transportation, home organization (e.g., cleaning and organizing the home), handling a crisis or medical emergency, financial responsibilities, or other.

- Neuropsychiatric Inventory Questionnaire (NPI-Q) [19] was completed by the CPD and used to ascertain neuropsychiatric symptoms in the care recipient. The NPI-Q is a 12-item respondent-administered questionnaire derived from the interview-based NPI [20] which assesses the presence/absence and severity (mild, moderate, severe) of behavioral and neurovegetative symptoms in the care recipient over the prior 4 weeks, and the resulting distress in the caregiver. The NPI-Q total score is the sum of individual symptom severity scores, ranging from 0 to 36. The caregiver distress score does not contribute to the overall total score.
- Caregiver Burden Inventory (CBI) [15] was used to assess caregiver burden. The CBI is a 24-item respondent-administered questionnaire with responses ranging from 0 (never) to 4 (almost always), and maximum total score of 84. It measures burden in 5 domains. The time-dependence domain encompasses burden due to the caregiver’s time being consumed by caring for the patient. Developmental burden relates to where the caregiver sees themselves in relation to their peers and where they envisioned they would be in their life in relation to their peers and their own life goals. Physical burden encompasses caregiver fatigue and health. The social burden domain of the CBI relates to the relationship of the caregiver to the care recipient and their family. Finally, emotional burden domain encompasses the feelings of the caregiver toward the care recipient, including embarrassment, shame, resentment, anger, or discomfort. All subdomains have 5 questions contributing to them except the physical domain which has 4.

### Statistical analysis

Sample characteristics, NPI-Q score, and CBI score and subscores were summarized with basic descriptive statistics [mean (SD) for continuous variables; frequencies and percentages for categorical variables].

Multivariable linear regression models were used to characterize the associations between NPI-Q total score (independent variables) and CBI total score or each of the 5 CBI domain subscores while adjusting for potential confounders. Covariates were selected based on their relationship with caregiver burden from the literature, namely caregiver age, sex, education, and caregiving duration [4, 6, 8, 21]. The same procedure was followed to model associations between NPI-Q individual symptom severity scores and the CBI total score as well as each of the 5 domain subscores. Scatterplots of the expected (fitted) values for CBI versus the actual (observed) values are included to illustrate how accurately caregiver burden may be predicted based on NPI symptoms.

All statistical analyses were performed using R version 3.6.3.

## Results

2740 individuals received the study invitation by email, and 741 clicked on the link to open the survey (Fig. 1). Compared to those who did not open the survey, those who did were older (64.9 vs 62.5 years, *p* < 0.001) and more likely to be male (29.4 male vs 20.6% male, *p* < 0.001, respectively). Among those who did not access the survey, data on age and sex were missing on 3 and 136, respectively.

The final sample was 450 individuals. Cohort characteristics are shown in Table 1. Mean age of the CPD was 65.87 (SD 10.39) years, and the majority were female (74%). Caregivers in this sample were predominantly spouses (84.9%), and 90.7% indicated they were the primary caregivers for the care recipient. Average duration of caregiving was 5.47 (SD 5.66) years. Half of the sample were full-time caregivers and 27% provided at least some daily care. Mean CBI total score was 31.73 (SD 17.66).

**Table 1:** Cohort characteristics

Variable	Result	
Age of caregiver, mean years (SD; range)	65.87 (10.39; 22.4–90.8)	
Sex M:F *N* (%)	117 (26): 333 (74)	
Education of caregiver *N* (%)		
Less than 9 years	5 (1.1)	
9–12 years	32 (7.1)	
13–16 years	240 (53.3)	
More than 16 years	173 (38.4)	
Relation of caregiver *N* (%)		
Spouse/partner	382 (84.9)	
Parent	46 (10.2)	
Sibling	8 (1.8)	
Uncle/aunt	0 (0)	
Employer	0 (0)	
Other	14 (3.1)	
Duration of caregiving, mean years (SD)	5.47 (5.66)	
Disease duration of care recipient, mean years (SD)	8.33 (6.43)	
Principal caregiver *N* (%)	408 (90.7)	
Employed outside the home *N* (%)	140 (31.1)	
Role of caregiver *N* (%)		
Not paid—lives with patient	396 (88)	
Not paid—doesn’t live with patient	48 (10.7)	
Paid and lives with patient	4 (0.9)	
Paid and doesn’t live with patient	2 (0.4)	
Time spent caregiving per week *N* (%) (*N* = 450)		
Full time	217 (48.2)	
A few hours a day every day	123 (27.3)	
A few days a week but not every day	44 (9.8)	
One day during the week or less	63 (14)	
Not answered	3 (0.7)	
Caregiver Burden Index, mean (SD)	31.73 (17.66)	
Caregiver Burden Subscore, mean (SD)		
Time	9.71 (5.42)	
Development	8.69 (5.35)	
Physical	6.12 (3.66)	
Emotional	3.49 (3.59)	
Social	3.72 (3.89)	
Caregiving responsibilities *N* (%)		
Assisting with personal care	202 (44.9)	
Food preparation	304 (67.6)	
Obtaining and/or administering prescribed medications	245 (54.4)	
General health care besides medications	302 (67.1)	
Mobility assistance	202 (44.9)	
Providing emotional support	416 (92.4)	
Transportation	310 (68.9)	
Home organization (e.g., cleaning and organizing the home)	333 (74.0)	
Handling a crisis or medical emergency	309 (68.7)	
Financial responsibilities	281 (62.4)	
Other (indoor/outdoor home repairs, caring for children and other family members)	89 (19.8)	

**Fig. 2:**
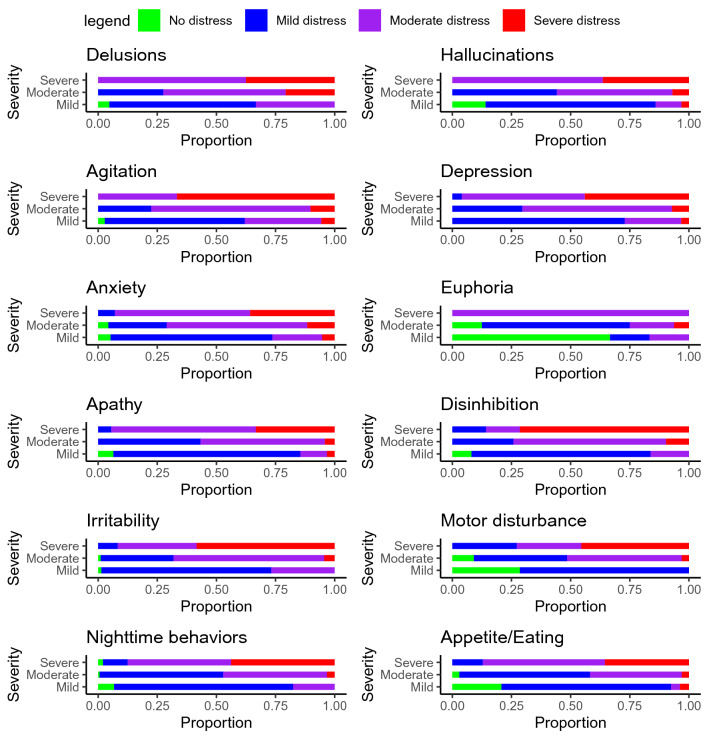
For each symptom on the NPI-Q, the proportion of caregivers reporting a given level of distress for a given severity of the symptom (in the care recipient) is shown (the mild distress category combines minimal and mild distress and the severe distress category combines severe and extreme distress)

An average of 3.63 (SD 2.63) symptoms in the care recipient were reported on the NPI-Q. NPI-Q total score was mean (SD) 6.33 (5.51). The most common neuropsychiatric symptoms reported were nighttime behaviors, depression, apathy, and irritability (Table 2). In general, severe symptoms in the care recipient (as rated by the CPD) were associated with moderate to severe distress in the care partner, but some also reported severe distress from mild symptoms or mild distress even with severe symptoms (Fig. 2).

**Table 2:** Prevalence of neuropsychiatric symptoms in care recipient by levels of symptom severity and caregiver distress

Measure	*N* (% of those with symptoms)	Distress *N* (% of those with specified symptom severity)					
		Not distressing	Minimal	Mild	Moderate	Severe	Extreme
Delusions (*N* = 58)							
Mild	21 (36.2)	1 (4.8)	4 (19)	9 (42.9)	7 (33.3)	0 (0)	0 (0)
Moderate	29 (50)	0 (0)	2 (6.9)	6 (20.7)	15 (51.7)	6 (20.7)	0 (0)
Severe	8 (13.8)	0 (0)	0 (0)	0 (0)	5 (62.5)	2 (25)	1 (12.5)
Hallucinations (*N* = 118)							
Mild	64 (54.2)	9 (14.1)	24 (37.5)	22 (34.4)	7 (10.9)	2 (3.1)	0 (0)
Moderate	43 (36.4)	0 (0)	8 (18.6)	11 (25.6)	21 (48.8)	3 (7)	0 (0)
Severe	11 (9.3)	0 (0)	0 (0)	0 (0)	7 (63.6)	4 (36.4)	0 (0)
Agitation (*N* = 135)							
Mild	71 (52.6)	2 (2.8)	17 (23.9)	25 (35.2)	23 (32.4)	4 (5.6)	0 (0)
Moderate	49 (36.3)	0 (0)	3 (6.1)	8 (16.3)	33 (67.3)	5 (10.2)	0 (0)
Severe	15 (11.1)	0 (0)	0 (0)	0 (0)	5 (33.3)	8 (53.3)	2 (13.3)
Depression (*N* = 215)							
Mild	92 (42.8)	0 (0)	23 (25)	44 (47.8)	22 (23.9)	3 (3.3)	0 (0)
Moderate	98 (45.6)	0 (0)	5 (5.1)	24 (24.5)	62 (63.3)	7 (7.1)	0 (0)
Severe	25 (11.6)	0 (0)	0 (0)	1 (4)	13 (52)	10 (40)	1 (4)
Anxiety (*N* = 140)							
Mild	57 (40.7)	3 (5.3)	22 (38.6)	17 (29.8)	12 (21.1)	3 (5.3)	0 (0)
Moderate	69 (49.3)	3 (4.3)	3 (4.3)	14 (20.3)	41 (59.4)	8 (11.6)	0 (0)
Severe	14 (10)	0 (0)	0 (0)	1 (7.1)	8 (57.1)	5 (35.7)	0 (0)
Euphoria (*N* = 23)							
Mild	6 (26.1)	4 (66.7)	0 (0)	1 (16.7)	1 (16.7)	0 (0)	0 (0)
Moderate	16 (69.6)	2 (12.5)	3 (18.8)	7 (43.8)	3 (18.8)	1 (6.2)	0 (0)
Severe	1 (4.3)	0 (0)	0 (0)	0 (0)	1 (100)	0 (0)	0 (0)
Apathy (*N* = 195)							
Mild	62 (31.8)	4 (6.5)	28 (45.2)	21 (33.9)	7 (11.3)	2 (3.2)	0 (0)
Moderate	97 (49.7)	0 (0)	7 (7.2)	35 (36.1)	51 (52.6)	4 (4.1)	0 (0)
Severe	36 (18.5)	0 (0)	1 (2.8)	1 (2.8)	22 (61.1)	11 (30.6)	1 (2.8)
Disinhibition (*N* = 75)							
Mild	37 (49.3)	3 (8.1)	16 (43.2)	12 (32.4)	6 (16.2)	0 (0)	0 (0)
Moderate	31 (41.3)	0 (0)	1 (3.2)	7 (22.6)	20 (64.5)	3 (9.7)	0 (0)
Severe	7 (9.3)	0 (0)	1 (14.3)	0 (0)	1 (14.3)	4 (57.1)	1 (14.3)
Irritability (*N* = 174)							
Mild	71 (40.8)	1 (1.4)	31 (43.7)	20 (28.2)	19 (26.8)	0 (0)	0 (0)
Moderate	91 (52.3)	1 (1.1)	4 (4.4)	24 (26.4)	58 (63.7)	4 (4.4)	0 (0)
Severe	12 (6.9)	0 (0)	0 (0)	1 (8.3)	4 (33.3)	5 (41.7)	2 (16.7)
Motor disturbance (*N* = 72)							
Mild	28 (38.9)	8 (28.6)	10 (35.7)	10 (35.7)	0 (0)	0 (0)	0 (0)
Moderate	33 (45.8)	3 (9.1)	1 (3)	12 (36.4)	16 (48.5)	1 (3)	0 (0)
Severe	11 (15.3)	0 (0)	2 (18.2)	1 (9.1)	3 (27.3)	5 (45.5)	0 (0)
Nighttime behaviors (*N* = 277)							
Mild	74 (26.7)	5 (6.8)	33 (44.6)	23 (31.1)	13 (17.6)	0 (0)	0 (0)
Moderate	155 (56)	1 (0.6)	34 (21.9)	47 (30.3)	68 (43.9)	5 (3.2)	0 (0)
Severe	48 (17.3)	1 (2.1)	1 (2.1)	4 (8.3)	21 (43.8)	18 (37.5)	3 (6.2)
Appetite/eating (*N* = 151)							
Mild	53 (35.1)	11 (20.8)	21 (39.6)	17 (32.1)	2 (3.8)	1 (1.9)	1 (1.9)
Moderate	67 (44.4)	2 (3)	14 (20.9)	23 (34.3)	26 (38.8)	2 (3)	0 (0)
Severe	31 (20.5)	0 (0)	0 (0)	4 (12.9)	16 (51.6)	9 (29)	2 (6.5)

The following regression results are presented as the change in expected value of CBI total or subscore per one-point increase in NPI-Q total score. In a linear regression model with CBI total score as the outcome and NPI total score as predictor, with caregiver age, sex, education, and duration of caregiving as covariates, the only variable associated with CBI total score was NPI total score (*β* = 1.957, *p* =  < 0.001, 95% CI 1.717–2.197); adjusted *R*^2^ = 39.2%.

For the contribution of severity of individual neuropsychiatric symptoms, Table 3 shows significant contributors in the linear regression models predicting CBI total score and each subscore; supplementary table 1 shows the full model.

**Table 3:** Significant neuropsychiatric and demographic contributors to domains of caregiver burden

Outcome	Predictors	Standardized *β* coefficient	*p* value	*β* coefficient	95% CI	Adjusted *R*^2^
CBI total score	Agitation severity	0.180	< 0.001	3.893	2.0, 5.786	0.399
	Anxiety severity	0.170	< 0.001	3.472	1.793, 5.151	
	Apathy severity	0.168	< 0.001	2.875	1.405, 4.345	
	Nighttime behaviors severity	0.118	0.005	1.957	0.581, 3.333	
	Hallucinations severity	0.109	0.025	2.531	0.318, 4.744	
	Depression severity	0.086	0.049	1.568	0.007, 3.129	
Time dependency	Anxiety severity	0.224	< 0.001	1.407	0.886, 1.928	0.385
	Hallucinations severity	0.172	< 0.001	1.228	0.540, 1.916	
	Female sex	− 0.133	0.001	− 1.642	− 2.591, − 0.693	
	Apathy severity	0.124	0.005	0.650	0.194, 1.106	
	Duration of caregiving	0.114	0.004	0.110	0.035, 0.185	
	Nighttime behaviors severity	0.098	0.022	0.500	0.073, 0.927	
	Age	0.088	0.027	0.046	0.005, 0.087	
Development	Apathy severity	0.217	< 0.001	1.120	0.660, 1.580	0.357
	Anxiety severity	0.169	< 0.001	1.047	0.522, 1.572	
	Nighttime behaviors severity	0.145	0.001	0.730	0.300, 1.160	
	Agitation severity	0.123	0.008	0.802	0.208, 1.396	
	Hallucinations severity	0.101	0.044	0.711	0.019, 1.403	
Physical	Nighttime behaviors severity	0.147	0.002	0.507	0.194, 0.820	0.275
	Anxiety severity	0.133	0.004	0.564	0.181, 0.947	
	Depression severity	0.131	0.006	0.497	0.141, 0.853	
	Apathy severity	0.112	0.021	0.396	0.062, 0.730	
	Agitation severity	0.096	0.05	0.432	0.002, 0.862	
Emotional	Disinhibition severity	0.218	< 0.001	1.201	0.641, 1.761	0.210
	Agitation severity	0.199	< 0.001	0.873	0.431, 1.315	
	Female sex	0.104	0.019	0.854	0.141, 1.567	
Social	Agitation severity	0.253	< 0.001	1.205	0.727, 1.683	0.212
	Age	− 0.193	< 0.001	− 0.072	− 0.105, − 0.039	
	Apathy severity	0.103	0.04	0.389	0.018, 0.760	

Six individual regression models were run, one for each respective CBI component, each containing all NPI severity items, age, sex, and caregiving duration. Full model shown in supplementary material

Significant contributors to the CBI total score, in order of greatest contribution [based on the magnitude of the standardized *β*-coefficient (s*β*)] were severity of agitation (s*β* = 0.180), anxiety (s*β* = 0.170), apathy (s*β* = 0.168), nighttime behaviors (s*β* = 0.118), hallucinations (s*β* = 0.109), and depression (s*β* = 0.086). Adjusted *R*^2^ for the model 39.9%.

Significant contributors to the time-dependency domain, in order of greatest contribution, were severity of anxiety (s*β* = 0.224), hallucinations (s*β* = 0.172), female sex (s*β* = − 0.133), severity of apathy (s*β* = 0.124), duration of caregiving (s*β* = 0.114), nighttime behaviors (s*β* = 0.098), and age (s*β* = 0.088). Adjusted *R*^2^ for the model was 38.5%.

Significant contributors to the developmental domain, in order of greatest contribution, were severity of apathy (s*β* = 0.217), anxiety (s*β* = 0.169), nighttime behaviors (s*β* = 0.145), agitation (s*β* = 0.123), and hallucinations (s*β* = 0.101). Adjusted *R*^2^ for the model was 35.7%.

Significant contributors to the physical domain, in order of greatest contribution, were severity of nighttime behaviors (s*β* = 0.147), anxiety (s*β* = 0.133), depression (s*β* = 0.131), apathy (s*β* = 0.112), and agitation (s*β* = 0.096). Adjusted *R*^2^ for the model was 27.5%.

Significant contributors to the emotional domain, in order of greatest contribution were severity of disinhibition (s*β* = 0.218), agitation (s*β* = 0.199), and female sex (s*β* = 0.104). Adjusted *R*^2^ for the model was 21.0%.

Significant contributors to the social domain, in order of greatest contribution were severity of agitation (s*β* = 0.253), age (s*β* = − 0.193), and apathy (s*β* = 0.103). Adjusted *R*^2^ for the model was 21.2%.

**Fig. 3:**
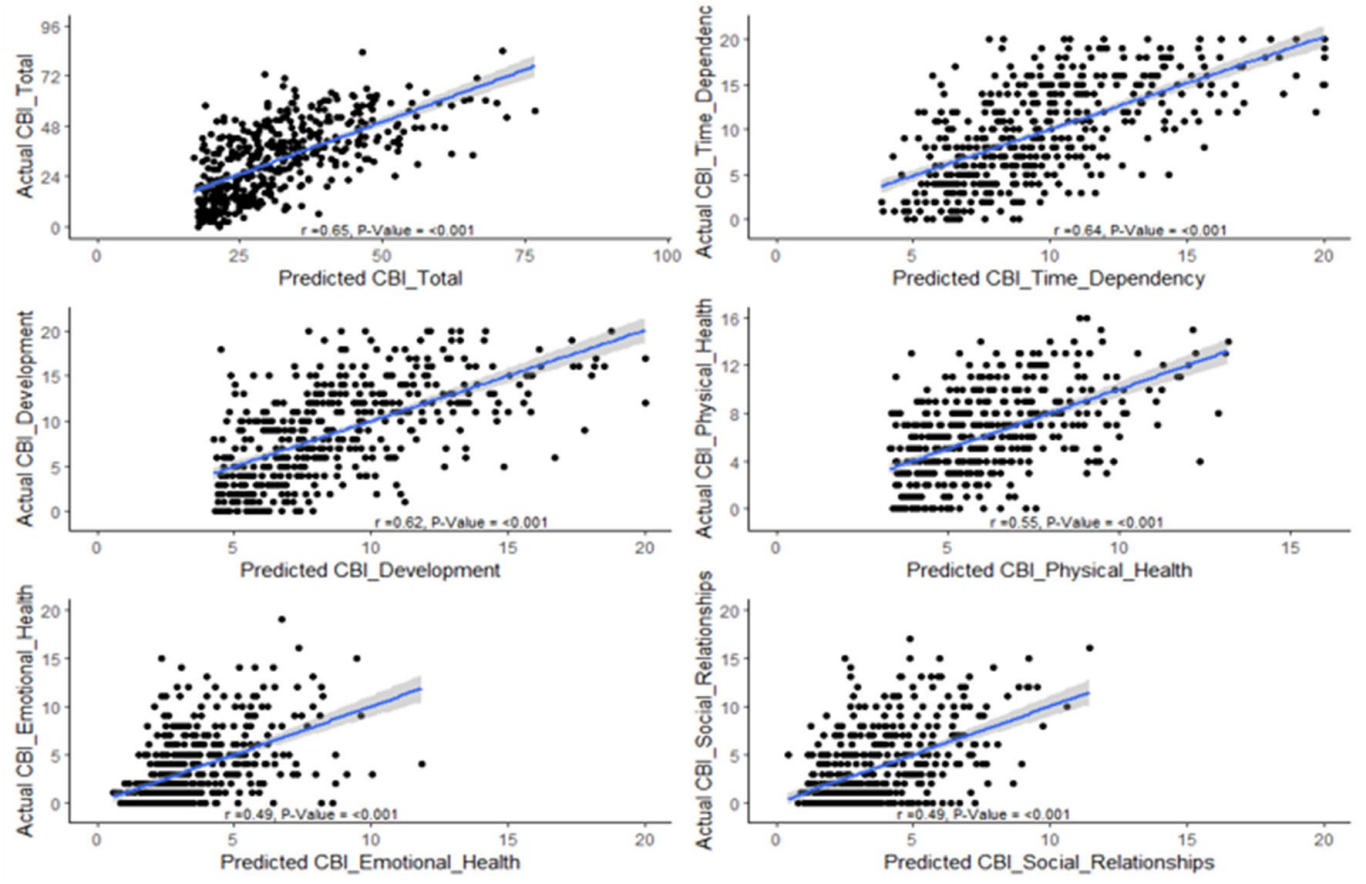
Plots of expected value of CBI scores (based on the regression models from Table 3) against the actual (observed) values

Figure 3 plots the expected value of CBI scores (based on the regression models from Table 3) against the actual (observed) values, providing a graphical representation of how much of the variation in CBI scores is explained by NPI-Q symptom severity. The positive associations for each plot (with correlation coefficients ranging from 0.49 to 0.65) suggest that NPI-Q symptoms explain a substantial amount of variation in the CBI scores, indicating that patient symptom severity is a strong contributor to caregiver burden.

## Discussion

Our findings highlight the complex, multidimensional, and multifactorial nature of caregiver burden in PD. Neuropsychiatric symptoms explained a substantial proportion of the variance in global caregiver burden, with anxiety, agitation, and apathy having the largest effect sizes. Importantly, specific neuropsychiatric symptoms and caregiver demographics contributed differentially to each domain of caregiver burden.

The time-dependence domain of caregiving relates to the extent to which the caregiver’s time is consumed by caring for the patient [15]. Each of the neuropsychiatric contributors to the time-dependence domain, namely hallucinations [22], anxiety [23], apathy [24], and nighttime behaviors [25] have been associated with greater disability and functional impairment in activities of daily living in PD. Thus, the caregiver of a patient with these neuropsychiatric symptoms may spend large amounts of time assisting and/or monitoring the patient, in turn allowing the caregiver less time for themselves. Indeed, the majority of caregivers in our sample reported providing care either full time or at least several hours every day. The most commonly reported responsibility, for 92% of caregivers, was the provision of emotional support to the patient. Consistent with this, anxiety severity in the care recipient had the largest effect size for the time-dependency domain of the CBI in the multivariable model. These findings raise at least three opportunities for intervention to improve time-dependency burden of caregiving for CPD: (1) identifying and treating anxiety and other neuropsychiatric symptoms in the care recipient, (2) providing education and tools to caregivers on how to address anxiety in the care recipient, and (3) providing respite care as a “break” from caregiving so that CPD may have time to themselves. Respite care may improve caregiver resilience and reduce caregiver burden [26]. Studies are needed to better understand the most useful means of delivering respite care for CPD, and in different settings.

Physical burden was influenced by apathy, anxiety, and nighttime behaviors, in addition to agitation. Greater functional dependence of the care recipient on the caregiver could be contributing to greater demands not only on the caregiver’s time but also on physical health. As with the time-dependency domain, anxiety had the largest effect size for the physical domain as well. Increased anxiety in the care recipient may prompt increased reliance on the caregiver, and in turn may even reduce the ability of the caregiver to maintain a support network [11]. This could reduce the direct or indirect respite care available to the caregiver [11], exacerbating physical burden. Behavioral and occupational therapy interventions to reduce time requirements as well as physical demands and exertion for caregiving in PD require study. A few randomized trials explored such interventions in PD [11], and did not demonstrate clear benefit.  However, the primary outcomes for these trials were global measures of caregiver burden [27, 28], and did not specifically examine the physical burden domain.

The construct of developmental burden encompasses the caregiver’s feelings regarding where they are in life compared to where they thought they would be or want to be [15]. Developmental burden has been associated with greater depression and lower caregiver satisfaction [29]. In our sample of CPD, it was influenced by hallucinations, anxiety, apathy, and nighttime behaviors, as well as agitation. Nighttime behaviors had the largest effect size. This is consistent with studies demonstrating a strong contribution of nocturnal symptoms in the care recipient to caregiver burden in PD [11, 30, 31]. This highlights not only the importance of treating nighttime symptoms in PD, but also the importance of incorporating caregiver burden outcome measures in any treatment intervention for nighttime symptoms in PD. Because developmental burden may be largely influenced by thought patterns and perceptions of the caregiver, counseling interventions designed to provide coping strategies to address this aspect of caregiver burden may also be useful [32]. This is also the case for emotional burden, which in the CBI relates to the caregiver’s feelings toward the care recipient.

Agitation and disinhibition were the main determinants of emotional burden, along with caregiver sex. This is consistent with data from other small studies of caregivers of dementia patients which indicated that feelings of resentment toward the care recipient were present among caregivers of patients with agitation and disinhibition [33, 34]. Importantly, a perception of willfulness [i.e., that the care recipient is behaving in an agitated and disinhibited manner willfully (“on purpose”)] is associated with caregiver resentment. The detection of emotional burden offers an opportunity to provide targeted education to caregivers toward the often-involuntary nature of agitation and disinhibition that occurs in, for example, PD dementia. Similarly, counseling interventions may be designed specifically to address the contribution of apathy and agitation to social burden. Apathy may influence social relationships, including between the caregiver and care recipient [35]. Preemptive caregiver education and support could reduce caregiver social isolation and loneliness [36, 37]. Several educational tools are available for PD patients and their caregivers, including materials developed via systematic approaches [38]. Controlled studies are needed, however, to determine the efficacy of these educational tools in alleviating caregiver burden and its specific domains.

Our findings indicate that there are demographic differences in different domains of caregiver burden in PD. Older age was associated with greater burden in the time-dependency domain, whereas younger age was associated with greater burden in the social domain. On the other hand, male sex was associated with greater time domain burden whereas female sex was associated with greater emotional domain burden. Little is known about sex differences in different caregiver burden domains in PD. Among older informal partner caregivers in the Netherlands, a similar pattern was noted [39], females were less likely than males to be burdened in the time domain, but had greater social burden. Factors behind these sex differences require further study. These results highlight the importance of tailored support programs for caregivers based on their age, sex, and relationship to the care recipient.

The symptoms assessed by the NPI-Q are strongly associated with cognitive dysfunction and dementia, though they can occur independently. The impact of psychosis symptoms on caregiver burden in PD may be greater when the care recipient has dementia [8]. Our study design did not allow us to determine cognitive function in the care recipient, and the mediation of the relationship between the neuropsychiatric symptoms, cognition, and CPD characteristics to each domain of caregiver burden requires study. Our study design also did not allow for an examination of motor severity and manifestations in the care recipient as a determinant of caregiver burden. While the severity of motor symptoms in the care recipient does influence caregiver burden, several studies have demonstrated a relatively minor contribution of motor disease severity to caregiver burden in PD compared to non-motor symptoms [8, 11, 30, 40]. In one study [41], the contribution of motor symptoms and 2 non-motor symptoms, depression and cognitive dysfunction, to spouse’s depression and strain was examined. Motor symptoms only explained 0–6% of the variance of caregiver strain compared to 7–13% explained by cognitive dysfunction/depression symptoms [41].

Regarding the means of assessment of neuropsychiatric symptoms in this study, caregivers’ report of neuropsychiatric symptoms in the care recipient has a strong established relationship with caregiver burden [6–11]. However, neuropsychiatric symptom burden in the care recipient as reported by the CPD is not always concordant with patient report or physician diagnosis [13, 14]. Indeed, caregivers may be more likely to report apathy and depression and less likely to report anxiety [14] and hallucinations [42]. However, it is notable that even when caregiver and physician/patient assessment of given neuropsychiatric symptoms is not concordant, caregiver *perception* of these symptoms still strongly influences caregiver distress and, importantly, caregiver distress may influence reporting of some symptoms in the care recipient as well [13, 14]. This has implications for design of caregiver support programs, emphasizing the importance of not only treating neuropsychiatric symptoms in the care recipient as appropriate, but also educating and otherwise supporting the caregiver in their own right.

The large sample size and the application of a multidomain caregiver burden questionnaire are noted strengths of this study. The strengths and limitations of using a caregiver-reported measure of neuropsychiatric symptoms in the care recipient have been discussed above. In addition, while the caregiver-reported NPI-Q shows strong concordance with the rigorously validated interviewer-administered NPI [43], online administration of the NPI-Q does not allow for the recommended clinician review of responses. Extension of our work, using other measures of neuropsychiatric symptoms—whether reported by the CPD, the patient, or the healthcare provider—will be important. The FI study does not currently allow the linking of caregiver-reported data and data reported by the care recipient but future work may introduce this functionality into the study, thus allowing for an examination of not only the relationship between caregiver burden and neuropsychiatric symptoms but also how the source of report (patient vs caregiver) influences this. As for other limitations, our sample consisted predominantly of female spouses, predominantly white, with relatively high levels of education. It is possible that these results are not generalizable to other informal CPDs, or formal (paid) CPD. In addition, as mentioned, cognitive function and motor symptoms in the care recipient were not examined.

Our findings emphasize the strong contribution of neuropsychiatric symptoms to caregiver burden in PD. They indicate that the domains of caregiving in PD are related to demographic characteristics of the caregiver and different neuropsychiatric symptoms in the care recipient. In light of our findings, interventions aimed at improving neuropsychiatric symptoms in PD that evaluate the effect on caregivers will benefit from assessing specific domains of caregiver burden. To optimally support CPD, management strategies may need to be tailored to each domain, its contributors and the characteristics of the CPD themselves.

## Supplementary Information

Below is the link to the electronic supplementary material.

Supplementary file1 (DOCX 85 KB)

## Data Availability

Data used in the preparation of this article are available on Fox Den at foxden.michaeljfox.org.

## Code availability

Code will be made available on request.
