# Supplementary material for: Contribution of neuropsychiatric symptoms in Parkinson’s disease to different domains of caregiver burden
Source: J Neurol. 2021 Feb 25;268(8):2961–72. doi: 10.1007/s00415-021-10443-7 (PMC8289810; doi:10.1007/s00415-021-10443-7)
Supplement: Supplementary file 1 — Supplementary file1 (DOCX 85 KB) [file 415_2021_10443_MOESM1_ESM.docx]

**Supplementary table 1.** Full model predicting CBI total score and each subscore.

| Outcome | Predictors | ß-Coefficient | 95% CI | Standardized ß Coefficient | P-Value | Adjusted R² |
| --- | --- | --- | --- | --- | --- | --- |
| CBI Total |  |  |  |  |  |  |
|  | (Intercept) | 25.573 | 9.429, 41.717 | 0 | 0.002 | 0.399 |
|  | Delusions Severity | 0.367 | -2.18, 2.914 | 0.013 | 0.777 |  |
|  | Hallucinations Severity | 2.531 | 0.318, 4.744 | 0.109 | 0.025 |  |
|  | Agitation Severity | 3.893 | 2, 5.786 | 0.18 | <0.001 |  |
|  | Depression Severity | 1.568 | 0.007, 3.129 | 0.086 | 0.049 |  |
|  | Anxiety Severity | 3.472 | 1.793, 5.151 | 0.17 | <0.001 |  |
|  | Euphoria Severity | -0.775 | -4.024, 2.474 | -0.018 | 0.639 |  |
|  | Apathy Severity | 2.875 | 1.405, 4.345 | 0.168 | <0.001 |  |
|  | Disinhibition Severity | 1.827 | -0.569, 4.223 | 0.068 | 0.135 |  |
|  | Irritability Severity | 0.531 | -1.287, 2.349 | 0.027 | 0.566 |  |
|  | Motor disturbance Severity | 1.134 | -0.945, 3.213 | 0.045 | 0.285 |  |
|  | Nighttime behaviors Severity | 1.957 | 0.581, 3.333 | 0.118 | 0.005 |  |
|  | Appetite/Eating Severity | 1.313 | -0.128, 2.754 | 0.072 | 0.074 |  |
|  | Age (years) | -0.012 | -0.144, 0.12 | -0.007 | 0.853 |  |
|  | Female | -0.959 | -4.013, 2.095 | -0.024 | 0.538 |  |
|  | Duration caregiving (years) | 0.173 | -0.065, 0.411 | 0.055 | 0.153 |  |
|  | Education:  9-12 years | -5.472 | -18.721, 7.777 | -0.08 | 0.417 |  |
|  | Education:  13-16 years | -6.309 | -18.721, 6.103 | -0.178 | 0.318 |  |
|  | Education:  More than 16 years | -5.427 | -17.837, 6.983 | -0.15 | 0.391 |  |
| CBI Time Dependency |  |  |  |  |  |  |
|  | (Intercept) | 5.333 | 0.317, 10.349 | 0 | 0.037 | 0.385 |
|  | Delusions Severity | 0.502 | -0.29, 1.294 | 0.06 | 0.213 |  |
|  | Hallucinations Severity | 1.228 | 0.54, 1.916 | 0.172 | <0.001 |  |
|  | Agitation Severity | 0.581 | -0.007, 1.169 | 0.088 | 0.053 |  |
|  | Depression Severity | 0.364 | -0.121, 0.849 | 0.065 | 0.141 |  |
|  | Anxiety Severity | 1.407 | 0.886, 1.928 | 0.224 | <0.001 |  |
|  | Euphoria Severity | -0.391 | -1.399, 0.617 | -0.029 | 0.447 |  |
|  | Apathy Severity | 0.65 | 0.194, 1.106 | 0.124 | 0.005 |  |
|  | Disinhibition Severity | -0.41 | -1.155, 0.335 | -0.049 | 0.28 |  |
|  | Irritability Severity | 0.123 | -0.441, 0.687 | 0.02 | 0.669 |  |
|  | Motor disturbance Severity | 0.456 | -0.191, 1.103 | 0.059 | 0.166 |  |
|  | Nighttime behaviors Severity | 0.5 | 0.073, 0.927 | 0.098 | 0.022 |  |
|  | Appetite/Eating Severity | 0.41 | -0.038, 0.858 | 0.074 | 0.072 |  |
|  | Age (years) | 0.046 | 0.005, 0.087 | 0.088 | 0.027 |  |
|  | Female | -1.642 | -2.591, -0.693 | -0.133 | 0.001 |  |
|  | Duration caregiving (years) | 0.11 | 0.035, 0.185 | 0.114 | 0.004 |  |
|  | Education:  9-12 years | -1.273 | -5.389, 2.843 | -0.06 | 0.544 |  |
|  | Education:  13-16 years | -1.343 | -5.197, 2.511 | -0.124 | 0.494 |  |
|  | Education:  More than 16 years | -1.506 | -5.36, 2.348 | -0.135 | 0.443 |  |
| CBI Development |  |  |  |  |  |  |
|  | (Intercept) | 5.415 | 0.36, 10.47 | 0 | 0.036 | 0.357 |
|  | Delusions Severity | -0.003 | -0.801, 0.795 | 0 | 0.995 |  |
|  | Hallucinations Severity | 0.711 | 0.019, 1.403 | 0.101 | 0.044 |  |
|  | Agitation Severity | 0.802 | 0.208, 1.396 | 0.123 | 0.008 |  |
|  | Depression Severity | 0.405 | -0.084, 0.894 | 0.073 | 0.104 |  |
|  | Anxiety Severity | 1.047 | 0.522, 1.572 | 0.169 | <0.001 |  |
|  | Euphoria Severity | -0.284 | -1.302, 0.734 | -0.022 | 0.584 |  |
|  | Apathy Severity | 1.12 | 0.66, 1.58 | 0.217 | <0.001 |  |
|  | Disinhibition Severity | 0.409 | -0.342, 1.16 | 0.05 | 0.285 |  |
|  | Irritability Severity | 0.152 | -0.418, 0.722 | 0.025 | 0.601 |  |
|  | Motor disturbance Severity | 0.182 | -0.469, 0.833 | 0.024 | 0.582 |  |
|  | Nighttime behaviors Severity | 0.73 | 0.3, 1.16 | 0.145 | 0.001 |  |
|  | Appetite/Eating Severity | 0.416 | -0.036, 0.868 | 0.076 | 0.071 |  |
|  | Age (years) | 0.012 | -0.029, 0.053 | 0.023 | 0.569 |  |
|  | Female | 0.343 | -0.614, 1.3 | 0.028 | 0.481 |  |
|  | Duration caregiving (years) | 0.007 | -0.068, 0.082 | 0.007 | 0.854 |  |
|  | Education:  9-12 years | -1.83 | -5.979, 2.319 | -0.088 | 0.386 |  |
|  | Education:  13-16 years | -1.812 | -5.698, 2.074 | -0.169 | 0.36 |  |
|  | Education:  More than 16 years | -1.312 | -5.198, 2.574 | -0.12 | 0.507 |  |
| CBI Physical Health |  |  |  |  |  |  |
|  | (Intercept) | 5.16 | 1.479, 8.841 | 0 | 0.006 | 0.275 |
|  | Delusions Severity | -0.045 | -0.627, 0.537 | -0.008 | 0.879 |  |
|  | Hallucinations Severity | 0.433 | -0.072, 0.938 | 0.09 | 0.092 |  |
|  | Agitation Severity | 0.432 | 0.002, 0.862 | 0.096 | 0.05 |  |
|  | Depression Severity | 0.497 | 0.141, 0.853 | 0.131 | 0.006 |  |
|  | Anxiety Severity | 0.564 | 0.181, 0.947 | 0.133 | 0.004 |  |
|  | Euphoria Severity | -0.218 | -0.959, 0.523 | -0.024 | 0.563 |  |
|  | Apathy Severity | 0.396 | 0.062, 0.73 | 0.112 | 0.021 |  |
|  | Disinhibition Severity | 0.251 | -0.295, 0.797 | 0.045 | 0.367 |  |
|  | Irritability Severity | 0.11 | -0.305, 0.525 | 0.027 | 0.602 |  |
|  | Motor disturbance Severity | 0.22 | -0.254, 0.694 | 0.042 | 0.361 |  |
|  | Nighttime behaviors Severity | 0.507 | 0.194, 0.82 | 0.147 | 0.002 |  |
|  | Appetite/Eating Severity | 0.229 | -0.099, 0.557 | 0.061 | 0.171 |  |
|  | Age (years) | -0.012 | -0.041, 0.017 | -0.033 | 0.442 |  |
|  | Female | -0.307 | -1.003, 0.389 | -0.037 | 0.386 |  |
|  | Duration caregiving (years) | 0.051 | -0.004, 0.106 | 0.079 | 0.064 |  |
|  | Education:  9-12 years | -0.838 | -3.859, 2.183 | -0.059 | 0.586 |  |
|  | Education:  13-16 years | -0.589 | -3.419, 2.241 | -0.08 | 0.683 |  |
|  | Education:  More than 16 years | -0.67 | -3.498, 2.158 | -0.089 | 0.642 |  |
| CBI Emotional Health |  |  |  |  |  |  |
|  | (Intercept) | 0.698 | -3.072, 4.468 | 0 | 0.716 | 0.21 |
|  | Delusions Severity | -0.199 | -0.795, 0.397 | -0.036 | 0.511 |  |
|  | Hallucinations Severity | 0.134 | -0.383, 0.651 | 0.028 | 0.611 |  |
|  | Agitation Severity | 0.873 | 0.431, 1.315 | 0.199 | <0.001 |  |
|  | Depression Severity | 0.047 | -0.317, 0.411 | 0.013 | 0.799 |  |
|  | Anxiety Severity | 0.204 | -0.187, 0.595 | 0.049 | 0.307 |  |
|  | Euphoria Severity | -0.19 | -0.949, 0.569 | -0.022 | 0.623 |  |
|  | Apathy Severity | 0.32 | -0.024, 0.664 | 0.092 | 0.068 |  |
|  | Disinhibition Severity | 1.201 | 0.641, 1.761 | 0.218 | <0.001 |  |
|  | Irritability Severity | -0.18 | -0.605, 0.245 | -0.045 | 0.406 |  |
|  | Motor disturbance Severity | 0.423 | -0.062, 0.908 | 0.083 | 0.087 |  |
|  | Nighttime behaviors Severity | 0.282 | -0.038, 0.602 | 0.083 | 0.085 |  |
|  | Appetite/Eating Severity | 0.153 | -0.183, 0.489 | 0.041 | 0.371 |  |
|  | Age (years) | 0.014 | -0.017, 0.045 | 0.039 | 0.381 |  |
|  | Female | 0.854 | 0.141, 1.567 | 0.104 | 0.019 |  |
|  | Duration caregiving (years) | -0.002 | -0.057, 0.053 | -0.003 | 0.954 |  |
|  | Education:  9-12 years | -0.522 | -3.616, 2.572 | -0.037 | 0.74 |  |
|  | Education:  13-16 years | -0.583 | -3.48, 2.314 | -0.081 | 0.693 |  |
|  | Education:  More than 16 years | 0.137 | -2.76, 3.034 | 0.019 | 0.926 |  |
| CBI Social Relationships |  |  |  |  |  |  |
|  | (Intercept) | 8.966 | 4.894, 13.038 | 0 | <0.001 | 0.212 |
|  | Delusions Severity | 0.112 | -0.531, 0.755 | 0.019 | 0.732 |  |
|  | Hallucinations Severity | 0.026 | -0.532, 0.584 | 0.005 | 0.928 |  |
|  | Agitation Severity | 1.205 | 0.727, 1.683 | 0.253 | <0.001 |  |
|  | Depression Severity | 0.255 | -0.138, 0.648 | 0.063 | 0.204 |  |
|  | Anxiety Severity | 0.251 | -0.172, 0.674 | 0.056 | 0.245 |  |
|  | Euphoria Severity | 0.308 | -0.512, 1.128 | 0.032 | 0.461 |  |
|  | Apathy Severity | 0.389 | 0.018, 0.76 | 0.103 | 0.04 |  |
|  | Disinhibition Severity | 0.377 | -0.228, 0.982 | 0.063 | 0.222 |  |
|  | Irritability Severity | 0.326 | -0.132, 0.784 | 0.075 | 0.163 |  |
|  | Motor disturbance Severity | -0.149 | -0.674, 0.376 | -0.027 | 0.578 |  |
|  | Nighttime behaviors Severity | -0.061 | -0.407, 0.285 | -0.017 | 0.731 |  |
|  | Appetite/Eating Severity | 0.104 | -0.26, 0.468 | 0.026 | 0.574 |  |
|  | Age (years) | -0.072 | -0.105, -0.039 | -0.193 | <0.001 |  |
|  | Female | -0.207 | -0.977, 0.563 | -0.023 | 0.597 |  |
|  | Duration caregiving (years) | 0.007 | -0.052, 0.066 | 0.01 | 0.826 |  |
|  | Education:  9-12 years | -1.008 | -4.351, 2.335 | -0.067 | 0.554 |  |
|  | Education:  13-16 years | -1.983 | -5.114, 1.148 | -0.255 | 0.214 |  |
|  | Education:  More than 16 years | -2.076 | -5.207, 1.055 | -0.26 | 0.193 |  |
